# Editorial: Myokines, Adipokines, Cytokines in Muscle Pathophysiology

**DOI:** 10.3389/fphys.2020.592856

**Published:** 2020-10-23

**Authors:** Valentina Di Felice, Dario Coletti, Marilia Seelaender

**Affiliations:** ^1^Department of Biomedicine, Neurosciences and Advanced Diagnostics, University of Palermo, Palermo, Italy; ^2^Sorbonne Université, CNRS, INSERM, Institut de Biologie Paris-Seine, IBPS, UMR 8256 Biological Adaptation and Ageing, Paris, France; ^3^Department of Anatomical, Histological, Forensic Sciences and Orthopedics, Sapienza University of Rome, Rome, Italy; ^4^Department of Anatomical, Histological, Forensic Sciences and Orthopedics, Interuniversity Institute of Myology, Rome, Italy; ^5^Department of Surgery, LIM26 HC, Medical School of the University of São Paulo, São Paulo, Brazil

**Keywords:** inflammation, myokines, skeletal muscle disease, organ cross talk, physical exercise

## Introduction

Individual striated muscle fibers communicate in both a paracrine and endocrine fashion and are also involved in the crosstalk with other tissues and organs such as the adipose tissue, immune system, liver, pancreas, bones, and brain (Delezie and Handschin, 2018). The striated muscle, which accounts for ~40% of body mass, presents high biosynthetic activity, and extensive vascularization, features that endorse current thinking that muscle is the largest endocrine system of the body (Benatti and Pedersen, 2015). There are hundreds of muscle secretory products, collectively known as myokines, including proteins, miRNA, and exosomes (Barone et al., 2016). Muscle secretion is significantly affected by muscle contraction (Son et al., 2018) due to the activation of mechanotransduction pathways (Coletti et al., 2016a). It has been suggested that the adipose tissue is also an endocrine organ, producing adipokines- leptin, and other hormones, in addition to cytokines (Galic et al., 2010). The inflammatory infiltrate in fat depots affects the course of several diseases, including cancer (Batista et al., 2012; Sawicka and Krasowska, 2016; Neto et al., 2018; Opatrilova et al., 2018), and an extensive review on the role of adipokines in disease has been published elsewhere (Orzechowski et al., 2014).

Myokines, adipokines, and cytokines are major therapeutic targets in both muscular and non-muscular diseases (Lindegaard et al., 2013; Manole et al., 2018), and understanding of their role in tissue crosstalk represents a subject of great interest in current biology. We have therefore chosen to address this paradigm within this Frontiers special issue on “Myokines, Adipokines, Cytokines in Muscle Pathophysiology.”

## Promoting Muscle Regeneration in Muscle Diseases

Inflammation impairs muscle regeneration, by affecting pro-myogenic genes, including NFAT5. In a cellular model of myositis, Herbelet et al. showed that impaired NFAT5 expression and nuclear translocation induced by the inflammatory cytokines IL-1 and INF-gamma. In patients with polymyositis and dermatomyositis, NFAT5 was unaffected, whereas in inclusion body myositis, NFAT5 was not expressed at all. This state is characterized by sub-clinical inflammation and oxidative stress is found in individuals consuming High Fat Diets (HFD) and contributes to the pathogenesis of the metabolic dysfunction observed in obesity. This has been demonstrated by Andrich et al. in rats consuming HFD for 14 days. HFD reduced GSH levels in the musculature, an indicator of altered antioxidant defense, but also, increased IL-6 gene expression. Rossi et al. reviewed the role of inflammation in skeletal muscle remodelling, highlighting that hypoxia and IL-6, upon strength training, augment macrophage and neutrophil recruitment.

HGF/c-met plays an important role in infiltrating macrophages during muscle regeneration (Choi et al.). HGF regulates macrophage transition from the inflammatory to the anti-inflammatory phenotype and acts on various cell types, including muscle progenitors, coordinating muscle regeneration.

It is noteworthy that severe conditions in which the muscle wastes away, such as cachexia, occur in the absence of overt local inflammation, and the atrophic muscle is not enriched in inflammatory cells (Berardi et al., 2008). Therefore, inflammatory cytokines in cachexia lead to protein breakdown and apoptosis, without establishing a chronic degeneration-inflammation cycle related to the detrimental action of local inflammatory cells (de Castro et al., 2019).

## Stem Cells to the Rescue

Stem cell-mediated muscle regeneration is regulated by circulating hormones and growth factors, by signals released by damaged muscle fibers, and by the extracellular matrix (Musarò et al., 2007). Myostatin (MST) and activin bind the activin receptor-2B (AcvR2B), with the effect of negatively regulating muscle growth and myogenesis (Lee and McPherron, 2001). Formicola et al. demonstrated that inhibition of the AcvR2B receptor rescues muscle regenerative potential, in parallel with the downregulation of ectopic fat deposition and fibrosis.

Muscle SC proliferation is affected *in vivo* by the expression of the histone deacetylase HDAC4, which acts as a transcriptional activator or repressor (Marroncelli et al., 2018). The paper by Renzini et al. demonstrated that the deletion of HDAC4 affects muscle regeneration and suggested that HDAC4 controls muscle regeneration *in vivo* via soluble factors.

Satellite cells are important, but not unique players in muscle regeneration. In another contribution, Biferali et al. review the role of fibro–adipogenic progenitors (FAPs) in muscle regeneration. After muscle injury, FAPs undergo massive expansion, followed by subsequent macrophage-mediated clearance. During this critical time window, FAPs establish a dynamic network of interactions supporting SC differentiation.

## Paracrine and Endocrine Mediators in Muscle Pathologies

A comprehensive review written by Penna et al. discusses the role of several pro-inflammatory cytokines produced by tumors and how they contribute to cachexia in cancer. Pro-cachectic factors also exert direct effects on muscle cells *in vitro*, as shown by Baccam et al., who demonstrated that tumor-derived-factors induce the activin-mediated atrophy of myotubes. A high level of circulating activin is an adverse prognostic factor in cancer patients (Loumaye et al., 2017), yet, the direct role of activin, beyond that of a marker of cachexia, has not been demonstrated. Follistatin, the physiological activin inhibitor produced by the myotubes upon mechanical stimulation, protects these cells from atrophy. These findings are in agreement with those reported in this same Frontiers special issue by Formicola et al..

The clinical relevance of muscle activity-dependent protective factors is highlighted in inactive patients, submitted to hemodialysis (HD), who show reduced levels of the beta-aminoisobutyric acid (BAIBA), a factor that has beneficial effects on muscle metabolism in an autocrine/paracrine manner. In HD patients, BAIBA levels were reduced in inactive individuals (Molfino et al.), confirming the importance of supplying nutritional support (Garcia et al., 2019) and/or exercise intervention in patients who have had long periods of physical inactivity.

Microgravity induces muscle atrophy in astronauts and Teodori et al. have investigated the role of miRNAs in the immune response to simulated microgravity *in silico*, revealing that microgravity induced conflicting signals that were responsible for muscle atrophy.

## Muscle as an Endocrine Organ

Over 600 myokines are released by the skeletal muscle (Görgens et al., 2015). The already long list of myokines keeps evolving, as new candidates are proposed to be classified into this category. This Frontiers special issue also includes several contributions on the subject, including a comprehensive review of the myokines released as a response to muscle contraction, by Lee and Jun. The potential of myokine to counteract muscle wasting under various conditions is further discussed by Piccirillo et al..

Adamo et al. have suggested that the skeletal muscle produces two hormones classically known as neurohypophyseal factors, i.e., vasopressin and oxytocin. The skeletal muscle may be both a source, as well as a target of these hormones. In a very provocative way, the findings reviewed by Adamo et al. challenge the classical view of endocrine glands as unique, anatomically defined sources of a given factor.

The skeletal muscle is important source of Fibroblast growth factor 21 (FGF21). Whether the action of one such factor is beneficial or detrimental for the muscle is still unclear, especially in humans. By examining the circulating levels of FGF21 in several physiological or pathological conditions we may gather further insight on its relevance and therapeutic value for treating metabolic diseases (Tezze et al.).

## Humoral Mediators of the Organ Cross-Talk

The perspective of white adipose tissue (WAT) has evolved from one in which it is a mere site for fatty acid storage and metabolism, to the current concept that WAT is a major endocrine organ with a specific role in the control of inflammation. Taking this into account, more recent studies have explored how adipocytes and WAT stromavascular components affect the striated muscle and a plethora of other tissues, especially in pathological conditions (Riccardi et al., 2020). Two articles in this issue address and discuss the crosstalk between muscle and adipose tissue: first a review on the myokine regulating both brown and white adipose tissue biology: myostatin, IL-6, IL-15, Irisin, FGF21, Angiopoietin-like protein (ANGPTL) and BAIBA (Leal et al.); second, a report on exercise-induced myokines release, affecting, adipokine release by WAT (Mika et al.).

It is well-established that exercise has beneficial effects on the heart. To highlight novel mechanisms within this context, Bellafiore et al. investigated the signaling molecules that regulate capillary growth in the healthy myocardium as a result of exercise. VEGFR-1/Flt-1, VEGFR-2/Flk-1, HIF-1α, and iNOS, act as myokines regulating angiogenesis in response to endurance training, consistent with the observed stimulation of capillary network development in healthy hearts, following physical exercise (Brown and Hudlicka, 2003).

The existence of organ crosstalk, involving multiple sources of the same molecule, such as of a specific cytokine, and the pleiotropic effects of many of these mediators, generate paradoxical effects and make it difficult to have a holistic view of the physiopathology of muscle, fat, and the immune system. An example of this complexity is IL-6, which is secreted by the muscle upon exercise but also by the immune cells and WAT during inflammation, inducing both hypertrophy and atrophy of the skeletal muscle while, at the same time, being responsive to exercise in the muscle and fat (Rosa Neto et al., 2009). An excellent review by Pedersen and Febbraio (2008) both sheds-light and reconciles different views regarding IL-6 and its role in the metabolism in health and disease. The full characterization of the specific signaling pathways activated in different organs and conditions is of pivotal importance for understanding the effects of cytokine in different tissues. The Janus kinase (JAK) or signal transducer and activator of transcription (STAT) pathway, is a key intracellular mediator of a variety of factors, including the IL-6 family (Moresi et al.). IL-6-mediated activation of the JAK/STAT pathway may have opposing effects, promoting muscle hypertrophy and satellite proliferation, on one hand, and favoring muscle wasting, on the other hand.

## Exercise Endocrinological Effects

It has been established that exercise plays a major role in the prevention of human diseases through endocrinological, metabolic, genetic, and even epigenetic mechanisms (Coletti et al., 2016b; Grazioli et al., 2017). However, not all types of exercise are equal. Hody et al. reviewed the benefits and the risks of eccentric contractions, such as downhill running. For its unique features, eccentric exercise has been proposed for innovative rehabilitative protocols, despite the fact—and actually for the very reason that—it is a muscle-damaging exercise protocol. Isaacs et al. have reported that the exertion caused by rhabdomyolysis induced plyometric exercise is associated with different extents of muscle damage and that it is related to pain in healthy subjects. This heterogeneity correlates with a selective increase of C-reactive protein, which should be considered when prescribing exercise for pathological conditions.

In addition to damage and inflammatory markers, exercise induces the release of extracellular vesicles from the muscle. Trovato et al. unveiled and extensively examined the role of vesicles in myokine delivery. The ways in which exercise-related myokines (exerkines) are involved in tissue crosstalk during physical exercise is a topic of interest and is likely to attract further research attention in the future. Exercise affects vasopressin and oxytocin expression in the neurons of the paraventricular nucleus (Farina et al., 2014) and induces a five-fold increase in the circulating levels of the neurohypophyseal hormone vasopressin (Melin et al., 1980). This increase is associated with the beneficial effects of exercise on muscle homeostasis and suggests a model whereby physical activity stimulates muscle secretion of the neurohypophyseal hormones, which increases muscle responsiveness to the same hormones through the up-regulation of their receptors (Adamo et al.). Taken together, this evidence reveals the molecular bases of the humoral crosstalk between muscles and the brain during exercise, reflecting the Latin motto *mens sana in corpore sano*, i.e., “a sound mind in a sound body.”

There are age-dependent differences in adaptation to exercise (Harber et al., 2012). Aged mice that are deficient in Nicotinamide Riboside Kinase 2 (NMRK2) have shown a maladaptive metabolic response to exercise in both types of striated muscle (Deloux et al.). These are not the first findings to indicate the age-dependent effects of exercise and training adaptation on muscle metabolism. For instance, young Serum Response Factor KO mice do adapt normally to endurance exercise (Djemai et al., 2019) despite the fact that these mice show muscle functional deficits with aging (Lahoute et al., 2008).

Mika, Macaluso et al. showed that exercise and linoleic acid change hepatic fatty acid composition by increasing n-3 polyunsaturated and branched chain fatty acid incorporation. These findings are particularly interesting in light of the beneficial effects of linoleic supplements in muscle pathology (Macaluso et al., 2012; Carotenuto et al., 2016a,b,c).

Taken together, the articles in this special issue demonstrate that myokines, adipokines, and cytokines are central players in muscle physiopathology. The striated muscle must, therefore, be studied and managed using multidisciplinary approaches to consider organ crosstalk and in order to propose state-to-the-art, tailored therapeutic interventions that encompass the full complexity and unique characteristics of each patient.

## Author Contributions

VDF, DC, and MS contributed equally to the writing of the paper and management of the special issue. All authors contributed to the article and approved the submitted version.

## Conflict of Interest

The authors declare that the research was conducted in the absence of any commercial or financial relationships that could be construed as a potential conflict of interest.

## References

[B1] Barone R. Macaluso F. Sangiorgi C. Campanella C. Marino Gammazza A. Moresi V.. (2016). Skeletal muscle Heat shock protein 60 increases after endurance training and induces peroxisome proliferator-activated receptor gamma coactivator 1 α1 expression. Sci. Rep. 6:19781. 10.1038/srep19781 26812922 PMC4728392

[B2] Batista M. L. Peres S. B. McDonald M. E. Alcantara P. S. Olivan M. Otoch J. P.. (2012). Adipose tissue inflammation and cancer cachexia: possible role of nuclear transcription factors. Cytokine 57, 9–16. 10.1016/j.cyto.2011.10.008 22099872

[B3] Benatti F. B. Pedersen B. K. (2015). Exercise as an anti-inflammatory therapy for rheumatic diseases-myokine regulation. Nat. Rev. Rheumatol. 11, 86–97. 10.1038/nrrheum.2014.193 25422002

[B4] Berardi E. Aulino P. Murfuni I. Toschi A. Padula F. Scicchitano B. M.. (2008). Skeletal muscle is enriched in hematopoietic stem cells and not inflammatory cells in cachectic mice. Neurol. Res. 30, 160–169. 10.1179/174313208X281046 18397608

[B5] Brown M. D. Hudlicka O. (2003). Modulation of physiological angiogenesis in skeletal muscle by mechanical forces: involvement of VEGF and metalloproteinases. Angiogenesis 6, 1–14. 10.1023/A:1025809808697 14517399

[B6] Carotenuto F. Albertini M. C. Coletti D. Vilmercati A. Campanella L. Darzynkiewicz Z.. (2016a). How diet intervention via modulation of DNA damage response through MicroRNAs may have an effect on cancer prevention and aging, an *in silico* study. Int. J. Mol. Sci. 17:752. 10.3390/ijms17050752 27213347 PMC4881573

[B7] Carotenuto F. Coletti D. Di Nardo P. Teodori L. (2016b). α-linolenic acid reduces TNF-induced apoptosis in C2C12 myoblasts by regulating expression of apoptotic proteins. Eur. J. Transl. Myol. 26:6033 10.4081/ejtm.2016.6033 28078067 PMC5220214

[B8] Carotenuto F. Costa A. Albertini M. C. Rocchi M. B. Rudov A. Coletti D.. (2016c). Dietary flaxseed mitigates impaired skeletal muscle regeneration: *in vivo, in vitro* and *in silico* studies. Int. J. Med. Sci. 13, 206–219. 10.7150/ijms.13268 26941581 PMC4773285

[B9] Coletti D. Aulino P. Pigna E. Barteri F. Moresi V. Annibali D.. (2016a). Spontaneous physical activity downregulates Pax7 in cancer cachexia. Stem Cells Int. 2016:6729268. 10.1155/2016/6729268 27034684 PMC4807049

[B10] Coletti D. Daou N. Hassani M. Li Z. Parlakian A. (2016b). Serum response factor in muscle tissues: from development to ageing. Eur. J. Transl. Myol. 26:6008. 10.4081/ejtm.2016.6008 27478561 PMC4942704

[B11] de Castro G. S. Simoes E. Lima J. D. C. C. Ortiz-Silva M. Festuccia W. T. Tokeshi F.. (2019). Human cachexia induces changes in mitochondria, autophagy and apoptosis in the skeletal muscle. Cancers 11:1264. 10.3390/cancers11091264 31466311 PMC6770124

[B12] Delezie J. Handschin C. (2018). Endocrine crosstalk between skeletal muscle and the brain. Front. Neurol. 9:698. 10.3389/fneur.2018.00698 30197620 PMC6117390

[B13] Djemai H. Hassani M. Daou N. Li Z. Sotiropoulos A. Noirez P.. (2019). Srf KO and wild-type mice similarly adapt to endurance exercise. Eur. J. Transl. Myol. 29:8205. 10.4081/ejtm.2019.8205 31354926 PMC6615070

[B14] Farina E. V. Cappello F. Lipari L. Valentino A. Di Felice V. Valentino B. (2014). Presence of oxytocin, vasopressin and atrial natriuretic peptide and their modification in rat hypothalamic paraventricular nucleus during resistance training. Anat. Histol. Embryol. 43, 159-163. 10.1111/ahe.12051 23551170

[B15] Galic S. Oakhill J. S. Steinberg G. R. (2010). Adipose tissue as an endocrine organ. Mol. Cell Endocrinol. 316, 129–139. 10.1016/j.mce.2009.08.018 19723556

[B16] Garcia M. Seelaender M. Sotiropoulos A. Coletti D. Lancha A. H. (2019). Vitamin D, muscle recovery, sarcopenia, cachexia, and muscle atrophy. Nutrition 60, 66–69. 10.1016/j.nut.2018.09.031 30529188

[B17] Görgens S. W. Eckardt K. Jensen J. Drevon C. A. Eckel J. (2015). Exercise and regulation of adipokine and myokine production. Prog. Mol. Biol. Transl. Sci. 135, 313–336. 10.1016/bs.pmbts.2015.07.002 26477920

[B18] Grazioli E. Dimauro I. Mercatelli N. Wang G. Pitsiladis Y. Di Luigi L.. (2017). Physical activity in the prevention of human diseases: role of epigenetic modifications. BMC Genomics 18(Suppl. 8):802. 10.1186/s12864-017-4193-5 29143608 PMC5688489

[B19] Harber M. P. Konopka A. R. Undem M. K. Hinkley J. M. Minchev K. Kaminsky L. A. (2012). Aerobic exercise training induces skeletal muscle hypertrophy and age-dependent adaptations in myofiber function in young and older men. J. Appl. Physiol. 113, 1495–1504. 10.1152/japplphysiol.00786.2012 22984247 PMC3524668

[B20] Lahoute C. Sotiropoulos A. Favier M. Guillet-Deniau I. Charvet C. Ferry A. (2008). Premature aging in skeletal muscle lacking serum response factor. PLoS ONE 3:e3910 10.1371/journal.pone.0003910 19079548 PMC2593784

[B21] Lee S. J. McPherron A. C. (2001). Regulation of myostatin activity and muscle growth. Proc. Natl. Acad. Sci. U.S.A. 98, 9306–9311. 10.1073/pnas.151270098 11459935 PMC55416

[B22] Lindegaard B. Hvid T. Grøndahl T. Frosig C. Gerstoft J. Hojman P. (2013). Expression of fibroblast growth factor-21 in muscle is associated with lipodystrophy, insulin resistance and lipid disturbances in patients with HIV. PLoS ONE 8:e55632 10.1371/journal.pone.0055632 23533568 PMC3606412

[B23] Loumaye A. de Barsy M. Nachit M. Lause P. van Maanen A. Trefois P.. (2017). Circulating Activin A predicts survival in cancer patients. J. Cachexia Sarcopenia Muscle 8, 768–777. 10.1002/jcsm.12209 28712119 PMC5659049

[B24] Macaluso F. Morici G. Catanese P. Ardizzone N. M. Gammazza A. M. Bonsignore G.. (2012). Effect of conjugated linoleic acid on testosterone levels *in vitro* and *in vivo* after an acute bout of resistance exercise. J. Strength Condit. Res. 26, 1667–1674. 10.1519/JSC.0b013e318231ab78 22614148

[B25] Manole E. Ceafalan L. C. Popescu B. O. Dumitru C. Bastian A. E. (2018). Myokines as possible therapeutic targets in cancer cachexia. J. Immunol. Res. 2018:8260742 10.1155/2018/8260742 30426026 PMC6217752

[B26] Marroncelli N. Bianchi M. Bertin M. Consalvi S. Saccone V. De Bardi M.. (2018). HDAC4 regulates satellite cell proliferation and differentiation by targeting P21 and Sharp1 genes. Sci. Rep. 8:3448. 10.1038/s41598-018-21835-7 29472596 PMC5823886

[B27] Melin B. Eclache J. P. Geelen G. Annat G. Allevard A. M. Jarsaillon E.. (1980). Plasma AVP, neurophysin, renin activity, and aldosterone during submaximal exercise performed until exhaustion in trained and untrained men. Eur. J. Appl. Physiol. Occup. Physiol. 44, 141–151. 10.1007/BF00421092 6997037

[B28] Musarò A. Giacinti C. Pelosi L. Dobrowolny G. Barberi L. Nardis C.. (2007). Stem cell-mediated muscle regeneration and repair in aging and neuromuscular diseases. Eur. J. Histochem. 51(Suppl. 1), 35–43. 17703592

[B29] Neto N. I. P. Murari A. S. P. Oyama L. M. Otoch J. P. Alcântara P. S. Figuerêdo M. J.. (2018). Peritumoural adipose tissue pro-inflammatory cytokines are associated with tumoural growth factors in cancer cachexia patients. J. Cachexia Sarcopenia Muscle 9, 1101–1108. 10.1002/jcsm.12345 30284380 PMC6240753

[B30] Opatrilova R. Caprnda M. Kubatka P. Valentova V. Uramova S. Nosal V.. (2018). Adipokines in neurovascular diseases. Biomed. Pharmacother. 98, 424–432. 10.1016/j.biopha.2017.12.074 29278852

[B31] Orzechowski A. Rostagno A. A. Pucci S. Chiocchia G. (2014). Cytokines and disease. Mediators Inflamm. 2014:209041. 10.1155/2014/209041 25301979 PMC4180395

[B32] Pedersen B. K. Febbraio M. A. (2008). Muscle as an endocrine organ: focus on muscle-derived interleukin-6. Physiol. Rev. 88, 1379-1406. 10.1152/physrev.90100.2007 18923185

[B33] Riccardi D. M. D. R. das Neves R. X. E. M. de Matos-Neto Camargo R. G. Lima J. D. C. Radloff K.. (2020). Plasma lipid profile and systemic inflammation in patients with cancer cachexia. Front. Nutr. 7:4. 10.3389/fnut.2020.00004 32083092 PMC7005065

[B34] Rosa Neto J. C. Lira F. S Oyama L. M Zanchi N. Yamashita A. S.. (2009). Exhaustive exercise causes an anti-inflammatory effect in skeletal muscle and a pro-inflammatory effect in adipose tissue in rats. Eur. J. Appl. Physiol. 106, 697–704. 10.1007/s00421-009-1070-1 19424714

[B35] Sawicka K. Krasowska D. (2016). Adipokines in connective tissue diseases. Clin. Exp. Rheumatol. 34, 1101–1112. 27463538

[B36] Son J. S. Chae S. A. Testroet E. D. Du M. Jun H. (2018). Exercise-induced myokines: a brief review of controversial issues of this decade. Expert. Rev. Endocrinol. Metab. 13, 51–58. 10.1080/17446651.2018.1416290 30063442

